# Burden and health-related quality of life of eating disorders, including Avoidant/Restrictive Food Intake Disorder (ARFID), in the Australian population

**DOI:** 10.1186/s40337-017-0149-z

**Published:** 2017-07-03

**Authors:** Phillipa Hay, Deborah Mitchison, Abraham Ernesto Lopez Collado, David Alejandro González-Chica, Nigel Stocks, Stephen Touyz

**Affiliations:** 1 Translational Health Research Institute (THRI), School of Medicine, Western Sydney University, Sydney, NSW Australia; 2 Centre for Emotional Health, Department of Psychology, Macquarie University, Sydney, Australia; 3 School of Medicine, Western Sydney University, Sydney, NSW Australia; 4 Centre for Health Research, School of Medicine, Western Sydney University, Sydney, NSW Australia; 5 Discipline of General Practice, Adelaide Medical School, NHMRC Centre of Research Excellence to Reduce Inequality in Heart Disease, The University of Adelaide, Adelaide, SA Australia; 6 School of Psychology, Faculty of Science, The University of Sydney, Camperdown, NSW 2006 Australia

**Keywords:** Binge eating disorder, Anorexia nervosa, Bulimia nervosa, Prevalence

## Abstract

**Background:**

Little is known about the epidemiology and health related quality of life (HRQoL) of the new DSM-5 diagnoses, Binge Eating Disorder (BED) and Avoidant/Restrictive Food Intake Disorder (ARFID) in the Australian population. We aimed to investigate the prevalance and burden of these disorders.

**Methods:**

We conducted two sequential population-based surveys including individuals aged over 15 years who were interviewed in 2014 (*n* = 2732) and 2015 (*n* =3005). Demographic information and diagnostic features of DSM-5 eating disorders were asked including the occurrence of regular (at least weekly over the past 3 months) objective binge eating ﻿with levels of distress﻿, extreme dietary restriction/fasting for weight/shape control, purging behaviors, overvaluation of shape and/or weight, and the presence of an avoidant/restrictive food intake without overvaluation of shape and/or weight. In 2014 functional impact or role performance was measured with the ‘days out of role’ question and in 2015, Health Related Quality of Life (HRQoL) was assessed with the Short Form −12 item questionnaire (SF-12v1).

**Results:**

The 2014 and 2015 3-month prevalence of eating disorders were: anorexia nervosa-broad 0.4% (95% CI 0.2–0.7) and 0.5% (0.3–0.9); bulimia nervosa 1.1% (0.7–1.5) and 1.2% (0.9–1.7); ARFID 0.3% (0.1–0.5) and 0.3% (0.2–0.6). The 2015 3-month prevalence rates were: BED-broad 1.5% (1.1–2.0); Other Specified Feeding or Eating Disorder (OSFED) 3.2 (2.6–3.9); and Unspecified Feeding or Eating Disorder (UFED) 10.4% (0.9–11.5). Most people with OSFED had atypical anorexia nervosa and majority with UFED were characterised by having recurrent binge eating without marked distress. Eating disorders were represented throughout sociodemographic groups and those with bulimia nervosa and BED-broad had mean weight (BMI, kg/m^2^) in the obese range. Mental HRQoL was poor in all eating disorder groups but particularly poor for those with BED-broad and ARFID. Individuals with bulimia nervosa, BED-broad and OSFED-Purging Disorder also had poor physical HRQoL. ARFID and bulimia nervosa groups had lower role performance than those without an eating disorder.

**Conclusions:**

Whilst full spectrum eating disorders, including ARFID, were less common than OSFED or UFED, they were associated with poor mental HRQoL and significant functional impairment. The present study supports the movement of eating disorders in to broader socio demographic groups including men, socio-economic disadvantaged groups and those with obesity.

## Plain English Summary

There are reports that feeding and eating disorders (FEDs) are becoming more common and in 2013 two new FEDs were introduced to Psychiatric practice, Avoidant/Restrictive Food Intake Disorder (ARFID) and Binge Eating Disorder (BED). Little is known about how common ARFID is or its impact on people’s lives compared to other established eating disorders like anorexia nervosa and bulimia nervosa. We conducted an interview survey of 5737 people aged over 15 years living in South Australia in 2014 or 2015. We asked questions about eating disorder symptoms like binge eating, purging, fasting and body image concerns and also how much their mental and physical health impacted on their ability to do what they wanted to do in their lives. We found about 1 in 200 people currently have anorexia nervosa, 1 in 100 people have bulimia nervosa, 1 in 300 people have ARFID and 1in 70 have BED. Most people with a FED did not meet full threshold criteria and had an Other Specified (OSFED) or Unspecified (UFED) type. OSFED and UFED groups were common, occurring in about 1 in 30 and 1 in 10 people respectively. People with FEDs came from all socio-economic groups and included many people who were overweight or obese. Except for people who had regular purging, many with OSFED or UFED reported little impact on their lives from mental illness. In contrast those with anorexia nervosa, bulimia nervosa, ARFID or BED reported very poor mental health related quality of life.

## Background

### Burden and health related quality of life of eating disorders in the Australian population

Eating disorder behaviours appear to be increasing in Australia and are associated with notable impact on individual’s health-related quality of life (HRQoL) [1]. Much less is known however about the general population burden of full syndrome eating disorders, and in particular the newly introduced Binge Eating Disorder (BED) and Avoidant/Restrictive Food Intake Disorder (ARFID) [2]. BED and ARFID differ from longer recognised disorders such as anorexia nervosa and bulimia nervosa in that they do not have a core psychopathology of body image disturbance or weight/shape overvaluation. BED is characterised by recurrent regular weekly episodes of uncontrolled over-eating (binge eating) associated with marked distress and the presence of three of five diagnostic specifiers (rapid, non-hungry, and solitary (due to embarrassment) eating, eating until uncomfortably full and/or depression/guilt/disgust after eating). ARFID is characterised by restricted food intake resulting in either macronutrient or micronutrient insufficiency, and not due to body image disturbance as in anorexia nervosa, or another mental or physical health disorder. The dietary restriction may be attributable to anxious food avoidance or a heightened sensitivity to the sight, smell or taste perception of food.

The diagnostic criteria of ARFID are a well-considered advance on a previously problematic area of classification of feeding problems in children [3]. However, the extension of ARFID from a disorder of childhood to a disorder that may occur throughout the lifespan was a notable departure in conceptualisation. There is some support for problematic childhood eating behaviours such as “picky” eating [4] to be present across all ages and to have more negative impact when co-occurring with eating disorder features such as weight/shape overvaluation, binge eating and weight control behaviours [5]. ARFID in adulthood is also conceptualised as a disorder that previously may have been diagnosed as an anxiety disorder, a specific phobia to a type of food or swallowing. In clinical settings it may be difficult to differentiate people with ARFID from those with anorexia nervosa who may minimise or deny the extent of weight/shape overvaluation, or in cultures with different expression of distress around body image and/or where mental illness is more commonly expressed through somatisation rather than psychological reactions [6, 7].

Whilst there is extensive empirical literature on the epidemiology of the classic eating disorders there is a paucity of accurate population data on the HRQoL and distribution of ARFID and BED. This is important to help understand the putative public health burden of these disorders and to inform potential need for health care services. Most studies of ARFID or like syndromes have reported its presence in paediatric samples where it may be up to a fifth of eating disorder presentations, around a third of paediatric gastroenterology presentations, and be more common in boys [8–10].

We have reported that in a South Australian sample in 2008 and 2009 the point prevalence over 3 months of anorexia nervosa and bulimia nervosa were under one per cent whereas the prevalence of BED syndromes were between five and six percent respectively [11]. This study used criteria of recurrent weekly binge eating in the absence of regular compensatory behaviours such as purging behaviours or extreme dieting or fasting to approximate BED. When the prevalence of recurrent binge eating was examined with the additional requirement of over-valuation of weight and/or shape (which has been suggested as a diagnostic specifier) the prevalence halved to around three percent. Further, the specifiers for binge eating as listed in the DSM-5 and the requirement for marked distress were not asked in this survey [11], which may have inflated the estimated prevalence [12]. A recent community study in the US using diagnostic specifiers reported much lower 3-month DSM-5 BED prevalence estimates of 1.19% (95% CI 1.04–1.37%) [13]. Similarly, a cohort study of women in mid-life [14] reported a prevalence of 1.03% (0.73–1.46) of DSM-5 BED.

The aims of the present study, therefore, were to extend previous research on the prevalence, burden and HRQoL of people with eating disorders in the South Australian population in new samples who were surveyed in 2014 and 2015. These surveys assessed features of ARFID and, in 2015, the diagnostic criteria of distress consequent to episodes of binge eating. Second aims were to explore the demographic and clinical features of individuals with DSM-5 eating disorders.

## Method

The Health Omnibus surveys reported in this paper were conducted in 2014 and 2015. The Health Omnibus is a survey conducted each year by Harrison Health Research under the auspices of the South Australian Health Commission and the University of Adelaide and the method of the present study replicated that of previous Health Omnibus surveys [15]. The survey is conducted using face-to-face interviews of a representative sample of the South Australian population. Interviews are respondent-based and ask a range of both demographic and health-related questions.

### Sample selection and recruitment

The sample selection and interview procedures were alike in 2014 and 2015. Metropolitan and rural “collector” districts in South Australia were selected systematically based on a probability proportional to size sampling procedure, according to the Australian Bureau of Statistics 2011 Census data. In a second stage, 10 dwellings were chosen systematically within each selected district. The person to be interviewed within each dwelling was the person who was 15 years or older and had their birthday most recently. The samples were non-replacement, and up to six visits were made to conduct an interview with the designated participant in each selected dwelling. Interviews were conducted from September until December in both years. In total 5737 people were interviewed. To ensure feasibility and participant understanding of questions, over 50 interviews were conducted in a pilot period during August of 2014 and 2015.

Of the 5,200 selected households in 2014, 2732 of 4,066 individuals eligible to participate completed the interview (participation rate: 67.2%). Of the 5,300 selected households in 2015, 3005 of 4,226 individuals eligible to participate completed the interview (participation rate: 71.1%). The majority reason for non-participation was refusal (*n* = 1323 in 2014 and *n* = 1220 in 2015).

### Ethics statement

Participants provide verbal rather than written informed consent in the Health Omnibus surveys. This is due to the practicalities of carrying out large-scale surveys and the low-risk nature of the survey content. The methods of both the 2014 and 2015 surveys were approved by the Human Research Ethics Committee of the University of Adelaide (H0972010). All participants provided verbal informed consent and if the respondent was aged 15–17 consent was signed by a parent or guardian.

### Assessment of eating disorder features

The interview included demographic questions on age, sex, household income and education, which were followed by diverse questions asking about the individual’s health status. The eating disorder questions were embedded towards the middle of the interview.

Five eating disorder features were assessed and then used to create the diverse eating disorder categories. The features included: binge eating (both objective and subjective); purging and extreme dietary restriction; overvaluation of weight or shape; and the presence of avoidant/restrictive food intake. The questions in the surveys that elicited information regarding the presence of the first four of these features were based on diagnostic questions from The Eating Disorder Examination [16], a structured interview used to assign eating disorder diagnosis.

In both surveys, objective binge eating was assessed by asking participants whether they regularly felt that they ate ‘*an unusually large amount of food*’ and at the same time experienced a feeling of being ‘*out of control*’. Purging was assessed by asking participants whether they regularly used laxatives, diuretics (water tablets), or self-induced vomiting as a means to control their weight or shape. Extreme dieting was assessed by asking participants whether they have regularly gone on a ‘*very strict diet’* or ‘*hardly eaten anything at all’* in order to influence their weight or shape. The term ‘regular’ used in each of these questions was defined as the behavior having occurred at least once per week over the 3 months before the interview. Participants were asked the level of importance they placed on weight and/or shape in determining their self-evaluation on a 6-point scale. Based on that question, participants were classified as either not having extreme weight and shape concerns (reported none to moderate importance of weight and shape in determining self-evaluation) or extreme weight and shape concerns (reported marked to extreme importance of weight and shape in determining self-evaluation). An avoidant/restrictive food intake was assessed with the question: *Are you currently avoiding or restricting eating any foods to the degree that you have lost a lot of weight and/or become lacking in nutrition (*e.g., *have low iron) and/or had problems with family, friends or at work?* Three affirmative possible answers were recorded: 1. *Yes - for cultural or medical reasons* e.g., *Lent, Ramadan, nut or other food allergy;* 2. *Yes - ‘dieting’ to prevent weight gain; and* 3. *Yes - any other reason* e.g.*, food dislike or fear of swallowing*. Only the third answer (e.g., dislike of or fear of swallowing) was considered for the diagnosis of ARFID (i.e., not due to weight/shape concerns, cultural reasons, or due to another medical condition). Current body mass index (BMI, kg/m^2^) data were calculated based on self-reported weight (kg) and height (m). In 2015, if participants endorsed any episodes of binge eating they were asked if the binge eating or overeating they experienced was usually associated with distress. Responses were coded as: *not at all; yes a little;* or *yes-a lot.* This criterion was considered as positive when the participants reported “a lot” of distress.

Two additional, non-diagnostic questions were asked in 2014. Participants were asked about subjective binge eating - a feeling of loss of control whilst eating an amount of food that is not unusually large [16]. For the primary purpose of another study investigating cardiovascular risk factors, participants were also asked the number of days in the past week they had done any vigorous activity for a total of at least 30 min, or any combination of moderate and/or vigorous physical activity for a total of at least 60 min (including 60 min of moderate exercise only).

### Derivation of diagnostic groups (Table 1)

**Table 1:** Derivation of current (three months) DSM-5 [2] diagnostic categories in the present study

Diagnosis	Features					Observations
	BMI	Strict dieting, fasting and/or purging	Weight/shape overvaluation (score ranged 0–6)	Binge eating episodes	Marked distress associated to binge eating	
Anorexia nervosa (AN)	<18.5	Weekly	≥4	Not required	n/a	DSM-5 criteria A, B and C
AN - broad	<18.5	Not required	≥4	Not required	n/a	DSM-5 criteria A and C
Bulimia nervosa (BN)	≥18.5	Weekly	≥4	Weekly	Not required	All DSM-5 criteria
BED -broad^a^	≥18.5	Not present	n/a	Weekly	Present	DSM-5 criteria A, D, E
OSFED - atypical AN^a^	≥18.5	Weekly	≥4	Not required	n/a	
OSFED - BN^a^	≥18.5	Weekly	≥4	Less than weekly	Not required	Bulimia nervosa of sub-threshold frequency. Duration <3-months not asked
OSFED - BED^a^	≥18.5	Not present	n/a	Less than weekly	Present	BED of sub-threshold frequency. Duration <3-months not asked
OSFED - purging disorder’^a^	≥18.5	Weekly	Not required	Not present	n/a	
Types of UFED^a^	≥18.5	Weekly purging	Not required	Not required	n/a	Not meeting criteria for any other eating disorder, including ARFID.
	≥18.5	Weekly diet/fasting	≥4	Not required	n/a	
	≥18.5	Not required	Not required	Weekly	n/a	
	≤18.5	Weekly	Not required	Not required	n/a/	
ARFID	Any	Not required	<4	Not required	n/a	Avoiding/restricting eating foods associated with weight loss and/or nutrition deficit and/or interpersonal problems, not for cultural or medical conditions.

^a^2015 only; *BED* Binge Eating Disorder, *OSFED* Other Specified Feeding or Eating Disorder, *UFED* Unspecified Feeding or Eating Disorder, *ARFID* Avoidant/Restrictive Food Intake Disorder

For the purpose of creating mutually exclusive DSM-5 [2] diagnostic categories of eating disorders, as summarised in Table 1, the previous features were combined as follows: anorexia nervosa was defined as participants with a BMI < 18.5, presence of strict dieting or fasting and/or purging for weight/shape control, and extreme weight/shape concerns (i.e., DSM-5 criteria A, B and C); anorexia nervosa-broad was defined as participants with BMI < 18.5 and extreme weight/shape concerns (i.e., DSM-5 criteria A and C); bulimia nervosa was defined as participants with weekly or more frequent objective binge eating episodes, and purging episodes or strict dieting occurring weekly, and extreme weight/shape concerns, and BMI ≥ 18.5 (i.e., all the DSM-5 criteria); BED - broad was defined as participants with weekly or more frequent objective binge eating episodes associated with “a lot” of distress, no weekly purging or restrictive dieting/fasting, and BMI ≥ 18.5 (i.e., DSM-5 criteria A, D, E).

OSFED ‘atypical anorexia nervosa’ was defined as a BMI ≥ 18.5, with regular dieting/fasting and/or purging, and extreme weight/shape concerns; OSFED bulimia nervosa-type (bulimia nervosa of sub-threshold frequency, noting duration less than 3-months was not asked) was defined as less than weekly objective binge eating episodes, purging or dieting/fasting weekly and extreme weight/shape concerns; OSFED BED-type (BED of sub-threshold frequency, noting duration less than 3-months was not asked) was defined as less than weekly objective bulimic episodes, no weekly purging or dieting, and not meeting criteria for anorexia nervosa; OSFED ‘purging disorder’ was defined as having weekly purging episodes, no objectively binge eating episodes and not meeting criteria for anorexia nervosa; and UFED was defined as having weekly objective binge eating episodes, *or* weekly purging episodes, *or* weekly strict dieting with extreme weight/shape concerns (as weekly strict dieting or fasting may fall within societal normative behavior for a person on a weight reduction diet), *or* weekly strict dieting/purging with BMI <18.5, and not meeting criteria for another diagnostic category.

ARFID was defined as (i) currently avoiding or restricting eating any foods to the degree that the person had either lost a lot of weight *and/or* become lacking in nutrition (e.g., have low iron) *and/or* had problems with family, friends or at work, *and* affirmed this was for “*any other reason* e.g., *food dislike or fear of swallowing*” *and not* for cultural or medical reasons e.g., Lent, Ramadan, nut or other food allergy or ‘dieting’ to prevent weight gain and (ii) no extreme weight/shape concerns.

Because of absent information in the 2014 survey on the distress associated with binge eating criterion for BED, the 2014 prevalence data pertain only to anorexia nervosa, anorexia nervosa-broad, bulimia nervosa and ARFID. In the 2014 analyses, these eating disorder groups are compared with people without regular weekly binge eating, purging or weekly strict dieting with extreme weight/shape concerns, or ARFID.

### Health-related quality of life and role performance

HRQoL was assessed in the 2015 survey with the Medical Outcomes Study Short Form (12-item), version 1 (SF-12v1). The 12 questions in this instrument evaluate HRQoL in the past four weeks, generating two scores ranging from 0 to 100 (physical and mental components), with higher values indicating a higher HRQoL [17, 18]). It has been used extensively in research interested in the impairment associated with physiological and psychiatric health conditions, and good psychometric properties have been demonstrated, including in an Australian population sample [19].

Role performance was assessed in the 2014 survey with a question modelled on an item employed by the National Comorbidity Studies [20] (Kessler & Frank, 1997): *During the past four weeks on how many days (approximately), if any, were you unable to complete your work, study or household responsibilities because of any problem with your physical or emotional health?* Data were recorded as a discrete variable, i.e., number of days. Previous research has indicated good convergent validity of this item with the subscales of the SF-12 [21].

### Data analysis

Data in both years were weighted by the inverse of the individual’s probability of selection, then re-weighted by age, sex and Local Government Area to benchmarks derived from the Australian Bureau of Statistics’ estimated Resident Populations in June 2013 and June 2014. Prevalence data are reported as percentages with 95% confidence intervals (C.I.) calculated using the Newcombe-Wilson [22] method without continuity correction using an Excel syntax. A unique categorical variable with multiple categories (no eating disorder, anorexia nervosa, bulimia nervosa, BED - broad, OSFED categories, UFED and ARFID) was created and then groups compared on demographic features (age, sex, and income) and clinical features (exercise levels, presence of regular subjective binge eating episodes and BMI) using one-way ANOVA with Tukey post-hoc tests for parametric data and Kruskal-Wallis, Mann-Whitney U and Chi-squared post-hoc (*χ* ^2^) tests as appropriate for ordinal or categorical data. Because of small numbers within OSFED groups, OSFED was analysed as one group. All statistical tests were performed using SPSS version 22.

## Results

### Three-month prevalence of DSM 5 disorders

As shown in Table 2, the 3-month prevalence of anorexia nervosa-broad, bulimia nervosa and ARFID were very similar in 2014 and 2015. No cases of anorexia nervosa were identified in either year. In both years people with bulimia nervosa were predominately non-purging (2014: 77%, 95% CI 59–88; 2015: 81%, 95% CI 66–91), i.e., compensated for binge eating through dietary restriction rather than vomiting or laxative use.

**Table 2:** Point (3-month) prevalence of bulimia nervosa and other DSM-5 eating disorders

		*n*	%	95% C.I.		
	2014			2015		
Anorexia Nervosa - broad	12	0.4%	0.2; 0.7	16	0.5%	0.3;0.9
Bulimia Nervosa	30	1.1%	0.7; 1.5	37	1.2%	0.9;1.7
BED-Broad	-	-	-	45	1.5%	1.1;2.0
ARFID	8	0.3%	0.1;0.5	10	0.3%	0.2;0.6
OSFED	n.a.			96	3.2%	2.6;3.9
Atypical AN				74	2.5%	2.0;3.1
Sub-threshold BN				14	0.5%	0.3;0.8
Subthreshold BED				11	0.4%	0.2;0.7
Purging Disorder				10	0.3%	0.2;0.6
UFED	n.a.			311	10.4%	0.9;11.5

Data reported in this table are weighted for South Australian norms, *BED* Binge Eating Disorder, *OSFED* Other Specified Feeding or Eating Disorder, *UFED* Unspecified Feeding or Eating Disorder, *ARFID* Avoidant/Restrictive Food Intake Disorder

In 2015, almost all of the 311 participants with UFED met the criteria on the basis of having at least weekly recurrent binge eating (*n* = 307, 99%), two participants who reported no binge eating met UFED criteria based on recurrent extreme dieting/fasting with extreme weight/shape concerns (however did not report BMI data and thus anorexia nervosa could not be ruled out), and two participants reported purging with less frequent binge eating and extreme weight/shape concerns. No participants met UFED criteria with weekly strict dieting and/or purging with BMI < 18.5.

In 2015, the prevalence of BED was similar to bulimia nervosa. The prevalence of recurrent binge eating (with or without distress) was however similar in both years; namely 10.1% (*n* = 277; 95% CI 9.0–11.3%) in 2014 and 13.0% (*n* = 390; 95% CI 11.8–14.2%) in 2015. In 2015, of people with bulimia nervosa, only 12 (32%) were distressed “a lot” about their binge eating episodes.

### Comparative demographic features between the eating disorder diagnostic groups

As shown in Table 3, there was an overall difference in mean age between the DSM-5 eating disorder groups in 2014 (*F*_df3,2336_ = 6.015, *p* < .001). Those with anorexia nervosa-broad and bulimia nervosa were significantly younger than people without an eating disorder (*p* < 0.05 in both cases).

**Table 3:** Comparative demographic and clinical features of participants with a DSM-5 eating disorder

	Age/years		Female sex		Exercise/days	Regular^#^ SBE	BMI	
	Median IQ range	Mean SD	*n %*		Median IQ range	*n*;% 95% C.I.	Mean SD	
Year assessed	2014	2015	2014	2015	2014	2014	2014	2015
Anorexia Nervosa- Broad	22*^a^	33.3^b^	13^a^	10^a^	2.1	0^a^	17.6^a^	17.9^b^
	17–57	16.5	62%;	63%	2.0–5.0	n.a.	0.7	0.5
Bulimia nervosa (BN)	35^a^	34.1^b^	20^a^	17^a^	3.8	17;56.7%^b^	28.1^b^	32.0^c^
	26–44	12.8	67%	46%	2.0–6.0	39.2–72.6	6.3	7.6
BED - Broad	n.a.	41.5	n.a.	31^a^	n.a.	n.a.	n.a.	32.8^c^
		16.3	n.a.	69%				8.7
ARFID	46	31.9	1^b^	5^a^	1.3	0^a^	25.9^b^	22.0^b^
	24–60	19.7	11%	50%	0–5.2	n.a.	4.9	3.9
OSFED	n.a.	46.1^a^	n.a.	67^b^	n.a.	n.a.	n.a.	27.8^e^
		18.1		70%			n.a.	6.3
Atypical AN	n.a.	43.5	n.a.	48	n.a.	n.a.	n.a.	27.8
		16.3		65%			n.a.	6.3
Sub-threshold BN	n.a.	34.4	n.a.	12	n.a.	n.a.	n.a.	26.7
		1.7		86%			n.a.	4.1
Year assessed	2014	2015	2014	2015	2014	2014	2014	2015
Sub-threshold BED	n.a.	44.4	n.a.	11	n.a.	n.a.	n.a.	28
		19.4		100%			n.a.	7.2
Purging Disorder	n.a.	67.5	n.a.	7	n.a.	n.a.	n.a.	26.9
		16.6		64%			n.a.	5.4
UFED	n.a.	38.3^b^	n.a.	141^c^	n.a.	n.a.	n.a.	27.1^e^
		16.0		45%				6.0
No eating disorder	47.0^b^	48.2^a^	1217^c^	1256^a^	3.0	30; 1.3%^a^	26.6^b^	26.8^a^
	31–63	19.3	51%	50%	1.0–6.0	1.0–1.9	5.5	5.7

Data reported in this table are weighted for South Australian norms, *BED* Binge Eating Disorder, *OSFED* Other Specified Eating or Feeding Disorder, *UFED* Unspecified Feeding or Eating Disorder, *SBE* Subjective binge eating episode

*data were skewed thus median and IQ range is presented; #at least weekly; data with differing superscripts are different on post hoc tests at *p* < .05

There was an overall difference in age between DSM-5 groups in 2015 (*F*_df_ = 18.98_6,2997_, *p* < 0.001) and post-hoc tests revealed that people with anorexia nervosa–broad, bulimia nervosa and UFED were significantly younger than people with OSFED or without an eating disorder (all *p* < 0.05).

In 2014 there was an overall difference between diagnostic groups for sex dispersion (*χ* ^2^ = 9.8, df = 3, *p* = 0.01). Post-hoc tests found there was a significantly higher proportion of men in the ARFID group and a significantly higher proportion of women in the anorexia nervosa-broad and bulimia nervosa groups, compared to people without these eating disorders (*p* < 0.05).

In 2015 there was an overall difference between diagnostic groups in regards to sex dispersion (*χ* ^2^ = 28.8, df = 6, *p* < 0.001). Post-hoc tests found people with anorexia nervosa-broad, bulimia nervosa, BED - broad and ARFID had a relatively even sex distribution that did not differ from people without an eating disorder. The sex distribution of OSFED did not differ from other eating disorder groups however women were over-represented in OSFED relative to participants without an eating disorder. Those with UFED was more likely to have an even sex distribution relative to other eating disorders (*p* < 0.05).

Yearly household income data were collated in 13 levels from “less than $A12,000” to “more than $180,000”. In 2014 there was an overall difference in income levels between diagnostic groups (K-W *χ* ^2^ = 11.0, df = 4, *p* = 0.01). Post-hoc tests (*p* < 0.05) showed that those with ARFID had significantly lower annual median household incomes ($20–30,000, IQ range $10–20,000; $30–40,000) than people without an eating disorder ($60–80,000; IQ range $40–50,000; $100–120,000) and compared to people with bulimia nervosa ($80–100,000; IQ range $40–50,000; 120–140,000). The median income for people with anorexia nervosa-broad was $50–60,000 (IQ range $12–20,000; 120–140,000).

In 2015 there was an overall difference in income levels between groups (K-W *χ* ^2^ = 26.5, df = 6, *p* < 0.001). Post-hoc tests (*p* < 0.05) showed that people with BED - broad had significantly lower income (median $40–50,000; IQ range 12–20,000; 120–140,000) than people without an eating disorder (median $60–80,000; IQ range $30–40,000; 100–120,000) and people with UFED (median $ 80–100,000, IQ range 70–80,000; 120–140,000). Those with UFED also had a higher income level than people with OSFED (median $60–80,000; $30–40,000; 100–120,000). People without an eating disorder (median $60–80,000; $30–40,000; 100–120,000) had lower incomes than people with UFED. Median income levels for people with anorexia nervosa-broad were: $50–60,000 (IQ range 30–40,000; 60–80,000), bulimia nervosa were $60–80,000 (IQ range $30–40,000; 80–10,000), and ARFID were $100–120,000 (IQ range $30–40,000; 100–120,000).

### Comparative clinical features between the eating disorder diagnostic groups

In 2014, there was an overall difference between groups in proportions of people with regular (at least weekly) subjective binge eating episodes (*χ* ^2^ = 389.2, df = 3, *p* < 0.001). Post-hoc tests revealed that participants with anorexia nervosa-broad, ARFID, and without an eating disorder reported fewer subjective binge eating episodes than those with bulimia nervosa (*p* < 0.05). Those with bulimia nervosa reported significantly more subjective binge eating episodes than all other participants (*χ* ^2^ = 141.1, df = 1, Fishers exact test *p* < 0.001). No one with anorexia nervosa - broad or ARFID reported regular subjective binge eating episodes.

No differences between diagnostic groups were found for level of exercise in 2014 (K-W *χ* ^2^ = 3.2, df = 3, *p* = 0.36). In 2014 there was an overall difference between groups in BMI (*F*_*df*_ = 16.9_4,2328_, *p* < 0.001). Post-hoc tests revealed that participants with anorexia nervosa-broad had significantly lower BMI than those with any other eating disorder or no eating disorder (*p* < 0.05). There were no significant differences in BMI observed between other eating disorder diagnostic groups.

In 2015, there was an overall difference between groups in BMI (*F*_*df*_ =22.2_6,2746_, *p* < .001). People with anorexia nervosa-broad and ARFID had a significantly lower BMI than participants with no eating disorder and other eating disorder groups (all *p* < 0.05). Bulimia nervosa and BED - broad participants did not differ in BMI (*p* > 0.05) however both had significantly higher BMI than those with no eating disorder and other eating disorder groups (all *p* < 0.05). Participants with OSFED and UFED did not differ in BMI (*p* > 0.05) but also had a significantly higher BMI than participants without an eating disorder (*p* < 0.05).

### Role performance and health-related quality of life

As shown in Table 4, in 2014, there were overall significant differences between groups in days unable to function (*χ* ^2^ = 14.7, df = 3, *p* = 0.002). People with bulimia nervosa and ARFID had more non-functional days than people without an eating disorder. No other differences between groups emerged.

**Table 4:** Role Performance, Mental and Physical Health Related Quality of Life (M/PHRQoL) of participants with a DSM-5 eating disorder

	Days out of role	MHRQoL	PHRQoL
	Median IQ range	Mean ISD	Mean SD
Year assessed	2014	2015	2015
Anorexia Nervosa- Broad	0	46.9	53.6^a,c^
	0–3	13.6	5.9
Bulimia nervosa (BN)	0^a^	44.8^b^	43.3^b,c^
	0–4	10.9	12.8
BED-Broad	n.a.	39.2^b^	42.1^b^
		11.5	11.6
ARFID	4.0^a^	38.7 ^b^	53.8^c^
	0–14	13.7	13.6
OSFED	n.a.	51.2	47.5
		8.8	11.6
Atypical AN	n.a.	51.5	48.4
		9.0	11.2
Sub-threshold BN	n.a.	50.2	49.0
		11.5	9.4
Sub-threshold BED	n.a.	51.1	49.9
		7.1	11.0
Purging Disorder	n.a.	38.5	39.3
		9.5	12.8
UFED	n.a.	51.8	50.3^a,c^
		9.0	8.6
No eating disorder	0^b^	52.8^a^	48.7^a,c^
	0–0	8.5	10.2

Data reported in this table are weighted for South Australian norms, *BED* Binge Eating Disorder, *OSFED* Other Specified Eating or Feeding Disorder, *UFED* Unspecified Feeding or Eating Disorder; data with differing superscripts are different on post hoc tests at *p* < .05

In 2015 there were overall significant differences in mental and physical HRQoL (*F*_*df*_ = 28.5_6,2976_, *p* < .001 *F*_*df*_ = 7.455_6,2976,_ *p* < .001 respectively. As shown in Table 4, participants with bulimia nervosa, BED - broad and ARFID had lower levels of mental HRQoL compared to participants without an eating disorder. Participants with bulimia nervosa and BED - broad had poorer physical HRQoL compared to participants without an eating disorder, anorexia nervosa-broad and UFED. Finally, participants with BED - broad had poorer physical HRQoL compared to participants with ARFID.

## Discussion

The present study found the 3-month community prevalence of DSM-5 anorexia nervosa-broad and bulimia nervosa to be similar to that reported in the 2008/2009 surveys [11]. BED was less frequent than expected and this was largely the effect of the added criterion of marked distress associated with binge eating episodes. Recurrent binge eating regardless of distress remained very common. ARFID, bulimia nervosa, BED-broad and purging subtypes of OSFED were the least common disorders. Whilst UFED was mostly comprised of people with recurrent binge eating without marked distress, the largest subtype of OSFED were people with atypical (normal weight) AN. If marked distress was required for binge eating episodes in BN, UFED would increase further in size.

A large minority of those with an eating disorder in both surveys were men. This supports previous surveys in South Australia and other research [23, 24]. Numbers of people with ARFID were too low to be confident about sex distribution and it cannot be concluded from this study that ARFID in adults is more likely to occur in adult males, as it is in male children [8, 9]. Similarly, the lower income levels of ARFID in 2014 were not found in 2015, where those with BED-broad had lowest income levels and people with UFED the highest. This is consistent with previous reports of the changing socio-demographic distribution of people with eating disorders into disadvantaged populations [25]. People with anorexia nervosa-broad or bulimia nervosa were younger than other eating disorder groups. This is consistent with known earlier ages of onset particularly in the case of anorexia nervosa [26].

All eating disorder groups characterised by recurrent binge eating had high levels of BMI with mean levels in the obese range for bulimia nervosa and BED - broad. This supports the findings of the 2008/9 surveys and other reports of the “changing weightscape” of bulimia nervosa [27]. However, it should be noted that the proportion of non-purging BN was much higher in this community sample than may be encountered in clinical samples, and purging, particularly self-induced vomiting, may be used in conjunction with or contribute to greater weight loss/weight suppression than exercise or dieting. It was unexpected that levels of exercise among eating disorder groups did not differ from the general population. Exercise may not be a common weight control method in Australians with an eating disorder and/or it may be that Australians are heeding health advice and exercise is becoming popular. Regular subjective binge eating was very common in people with bulimia nervosa. This supports its consideration as an alternate or additional form of binge eating in the ICD-11 diagnostic classification schemes [28].

While less common, the current (monthly) role performance of people with bulimia nervosa and ARFID was impaired and mental health HRQoL poorer for those with bulimia nervosa, BED-broad and ARFID. Furthermore, physical HRQoL was poorer people with bulimia nervosa and BED-broad. The very low levels of HRQoL for BED-broad, lower than other eating disorders, has not been found in other studies [29]. This likely is further support for the validity of the distress criterion as it is delineating a group with profound health effects from their eating disorder. The poor physical health may be due to the consequences of comorbid weight disorder, with obesity common in BED. Physical health may also be impacted in bulimia nervosa from purging behaviours (as it was in OSFED purging disorder type), although purging was uncommon among participants with bulimia nervosa in this study. Further studies are needed to delineate the reasons for poor physical health in EDs of normal or overweight individuals, however even behaviours such as dietary fasting and overall food restriction are expected to have physical side effects such as fatigue. Further, ARFID is associated with nutritional deficiencies, which may be expressed as fatigue or other physical symptoms. The relatively unimpaired HRQoL for OSFED (excepting purging disorder) and UFED casts some doubt on the clinical significance of these eating disorders and whether they should be regarded as subclinical eating disorders rather than (as in the former EDNOS category) subthreshold disorders.

Limitations of the present study include the survey questions. There are no validated instruments for assessing features of ARFID and it was not possible to further explore the reasons for the avoidant/restrictive eating or to explore a greater variety of reasons for food avoidance e.g., sensory aversion to the sights/texture of foods, or the fear of food contamination of different foods when they touch one another while on the plate. It is possible that people identified as having ARFID may have in fact had no eating disorder or another eating disorder if, for example, a follow-up interview could have established that the under-eating was due to another cause such as insufficient income to purchase food or to build musculature (although people with such problems as muscle dysmorphic disorder would likely endorse weight/shape overvaluation [30] which is an exclusionary criteria for ARFID). In addition, the weight control behaviors used at less than weekly frequency were not determined and people with predominately subjective rather than objective binge eating who may have been present in the UFED category (or ICD-11 proposed bulimia nervosa and BED) were not identified. The use of a respondent-based interview may have contributed to over-reporting of frequency of binge eating. In addition, eating disorders characterised by compulsive exercise and night eating syndrome were not assessed. Finally, lower numbers in some groups, especially anorexia nervosa-broad and ARFID likely explain the inconsistent findings between the 2014 and 2015 surveys and may have contributed to Type II error in secondary analyses of between group differences. Thus caution should be applied in the interpretation of these results.

## Conclusions

In these surveys full syndrome eating disorders were uncommon in the general community but were associated with marked impairments in physical and mental HRQoL and role performance. Similar to anorexia nervosa, it may not be possible to undertake detailed and conclusive studies of ARFID in the community as it is difficult to detect cases, or else much larger samples are required. However, the conceptualization of ARFID in this study was supported by the finding that its diagnosis has an impact on mental HRQoL. The study supports others which have found men to comprise a large minority of those with eating disorders. Further studies are needed to assess the utility of the diagnostic criterion of subjective binge eating for bulimia nervosa and BED.
