# Correction: Burden and health-related quality of life of eating disorders, including Avoidant/Restrictive Food Intake Disorder (ARFID), in the Australian population

**DOI:** 10.1186/s40337-023-00909-6

**Published:** 2023-11-13

**Authors:** Phillipa Hay, Deborah Mitchison, Abraham Ernesto Lopez Collado, David Alejandro González-Chica, Nigel Stocks, Stephen Touyz

**Affiliations:** 1 Translational Health Research Institute (THRI), School of Medicine, Western Sydney University, Sydney, NSW Australia; 2 Centre for Emotional Health, Department of Psychology, Macquarie University, Sydney, Australia; 3 School of Medicine, Western Sydney University, Sydney, NSW Australia; 4 Centre for Health Research, School of Medicine, Western Sydney University, Sydney, NSW Australia; 5 Discipline of General Practice, Adelaide Medical School, NHMRC Centre of Research Excellence to Reduce Inequality in Heart Disease, The University of Adelaide, Adelaide, SA Australia; 6 School of Psychology, Faculty of Science, The University of Sydney, Camperdown, NSW 2006 Australia

**Correction: Journal of Eating Disorders (2017) 5:21** 10.1186/s40337-017-0149-z

The original publication [1] of this article contained an incomplete footnote for Table 2. The footnote in the original article read, “Data reported in this table are weighted for South Australian norms, BED (Binge Eating Disorder), OSFED (Other Specified Feeding or Eating Disorder), UFED (Unspecified Feeding or Eating Disorder), ARFID (Avoidant/Restrictive Food Intake Disorder).”

The complete footnote should have been, “Data reported in this table are weighted for South Australian norms, BED (Binge Eating Disorder), OSFED (Other Specified Feeding or Eating Disorder), UFED (Unspecified Feeding or Eating Disorder), ARFID (Avoidant/Restrictive Food Intake Disorder). Please note that the OSFED prevalence estimates are not mutually exclusive categories. Thirteen people with Bulimia Nervosa also met criteria for Atypical Anorexia Nervosa.”

The original article [1] has been corrected.
